# Optimized beamforming for simultaneous MEG and intracranial local field potential recordings in deep brain stimulation patients

**DOI:** 10.1016/j.neuroimage.2009.12.115

**Published:** 2010-05-01

**Authors:** Vladimir Litvak, Alexandre Eusebio, Ashwani Jha, Robert Oostenveld, Gareth R. Barnes, William D. Penny, Ludvic Zrinzo, Marwan I. Hariz, Patricia Limousin, Karl J. Friston, Peter Brown

**Affiliations:** a UCL Institute of Neurology, Queen Square, WC1N 3BG, London, UK; b Donders Institute for Brain, Cognition and Behaviour, Radboud University, Nijmegen, The Netherlands

## Abstract

Insight into how brain structures interact is critical for understanding the principles of functional brain architectures and may lead to better diagnosis and therapy for neuropsychiatric disorders. We recorded, simultaneously, magnetoencephalographic (MEG) signals and subcortical local field potentials (LFP) in a Parkinson's disease (PD) patient with bilateral deep brain stimulation (DBS) electrodes in the subthalamic nucleus (STN). These recordings offer a unique opportunity to characterize interactions between the subcortical structures and the neocortex. However, high-amplitude artefacts appeared in the MEG. These artefacts originated from the percutaneous extension wire, rather than from the actual DBS electrode and were locked to the heart beat. In this work, we show that MEG beamforming is capable of suppressing these artefacts and quantify the optimal regularization required. We demonstrate how beamforming makes it possible to localize cortical regions whose activity is coherent with the STN-LFP, extract artefact-free virtual electrode time-series from regions of interest and localize cortical areas exhibiting specific task-related power changes. This furnishes results that are consistent with previously reported results using artefact-free MEG data. Our findings demonstrate that physiologically meaningful information can be extracted from heavily contaminated MEG signals and pave the way for further analysis of combined MEG-LFP recordings in DBS patients.

## Introduction

Deep brain stimulation (DBS) is a method for treatment of some neurological and psychiatric disorders by electrical stimulation of subcortical brain structures through chronically implanted macro-electrodes. DBS has been especially effective for treating symptoms of Parkinson's disease (PD) and dystonia. It is also investigated in surgical treatment of Tourette syndrome, chronic pain, cluster headache, obsessive compulsive disorder, depression, epilepsy, and minimally conscious states (Awan et al., 2009). The brain structures targeted by DBS include the subthalamic nucleus (STN), globus pallidus, anterior cingulate, various nuclei of the thalamus, nucleus accumbens, anterior capsule, ventral caudate, and the brainstem (Awan et al., 2009). For a researcher, DBS offers a unique opportunity to record signals from structures that are not easily accessible with non-invasive electrophysiological methods (Hammond et al., 2007). Such recordings are sometimes performed in the operating room during surgery to optimize electrode placement. They can also be performed outside the operating theatre, when the DBS electrodes are externalized via a percutaneous extension wire. In the latter case, it is possible to record local field potentials (LFP) from the four contacts of the DBS electrode (Williams et al., 2003). The main advantage of working with patients with externalized electrodes is that it is possible to perform studies in laboratory settings, while patients are fully alert and without the stress of the operation, and, if required, under different pharmacological states.

There are many studies of the changes in the LFP associated with performance of various tasks, from self-initiated simple movements to complex cognitive experiments (Lalo et al., 2008; Fogelson et al., 2006; Cassidy et al., 2002; Williams et al., 2002; Purzner et al., 2007; Androulidakis et al., 2007; Kempf et al., 2007; Brown et al., 2006; Kühn et al., 2006; Amirnovin et al., 2004; Paradiso et al., 2004; Williams et al., 2003). Some of these studies also involved simultaneous acquisition of LFP and surface electroencephalography (EEG) (Lalo et al., 2008; Fogelson et al., 2006; Cassidy et al., 2002; Williams et al., 2002). This multimodal approach makes it possible to investigate the interactions between deep brain structures targeted by DBS and other (usually cortical) sources, whose activity is expressed in the EEG. Initial investigations suggested that activity in deep areas may be coupled to the cortical activity in two frequency ranges, referred to as the beta and the gamma bands (Lalo et al., 2008; Fogelson et al., 2006; Cassidy et al., 2002; Williams et al., 2002). The precise balance of coupled activities varies during task performance and depends on pharmacological state (Lalo et al., 2008; Fogelson et al., 2006; Cassidy et al., 2002; Williams et al., 2002).

EEG has the advantages of being relatively inexpensive, mobile and prevalent in the clinical setting. However, it also suffers from two severe limitations when applied to DBS patients. Since DBS implantation patients usually have sutures and bandages, it is not possible to apply more than a few electrodes to the scalp, which precludes a complete picture of cortical activity. In addition, burr holes in the patient's skull change its conductivity properties and render standard EEG forward models used for source localization inapplicable (Oostenveld and Oostendorp, 2002; Bénar and Gotman, 2002). By contrast, magnetoencephalography (MEG) can be recorded with a large number of sensors placed in a helmet-shaped array around the head without direct skin contact. Since the skull is transparent to magnetic fields, MEG forward models are less affected by burr holes and are in general more precise than EEG forward models; as they do not involve difficult to measure conductivity parameters (Van den Broek et al., 1998). In summary, simultaneous recording of MEG and intracranial LFP afford the potential to localize precisely cortical areas interacting with the deep brain structures targeted for stimulation. When such areas are far enough apart, MEG allows one to separate the activities from those areas and therefore study the behaviour of distributed brain networks in humans.

We have recently completed a series of simultaneous LFP-MEG recordings in PD patients with DBS electrodes in the STN. One major hurdle we faced when analyzing these data was the presence of high-amplitude artefacts in the MEG recording. In the present paper we report our findings concerning the origin and properties of these artefacts and describe a method to suppress them using MEG beamforming. We apply this method to data from a single patient and demonstrate several possible applications for subsequent data analysis.

This is the first report of combined subcortical LFP and MEG recording with the purpose of characterizing oscillatory activity in subcortico-cortical loops. In several previous studies (Kringelbach et al., 2007, Makela et al., 2007, Ray et al., 2009) MEG was recorded in DBS patients with implanted stimulators to examine the effect of stimulation on cortical activity. This is an interesting scientific question (that we have not addressed), to which our methodology may be applicable.

In what follows, we test the hypothesis that beamforming is particularly suitable for suppressing artefacts that confound MEG recordings of DBS patients. This rests on the assumption that the metal artefacts cannot be explained by localized cortical sources and can, in principle, be suppressed by beamforming. Beamforming has been shown analytically to be superior to conventional dipole analysis for suppressing external interferences (Brookes et al., 2008a). It has also been demonstrated (Cheyne et al., 2007) that beamforming is capable of source localization in the presence of severe metal artefacts, in subjects with metallic orthodontic implants or fillings. This speaks to the utility of beamforming in suppressing artefact of this sort. Beamforming is based on a conventional forward model of electromagnetic responses and the sample covariance matrix in sensor space. It furnishes estimates of source activity based on the assumption that data are caused by temporally uncorrelated sources. Highly regularized solutions correspond to the assumption that all the data are caused by one source, while decreasing the regularization suppresses contributions from other putative sources (Brookes et al., 2008b). We therefore tried to establish the nature of the artefact and confirm that it could be suppressed efficiently using beamforming. Artefact characterization was validated against the real patient analyses. Having established that the artefact could not confound beamformer estimation (due to its non source-like sensor topography) we optimized the degree of regularization. This was achieved by quantifying the variance in reconstructed source activity attributable to the artefact over different levels of regularization. This allowed us to identify a sufficient range of regularization for artefact suppression. To establish the face-validity of beamforming in this context, we examined reconstructed source activity looking for canonical time–frequency correlates of movement-related activity. Finally, we addressed the predictive validity of these estimates using their reproducibility over independent estimates of time–frequency responses.

## Methods

### Patient and surgery

Most data reported in this study were recorded from a patient (48-year-old left-handed male) with severe PD, who underwent implantation of DBS electrodes in the STN. The study was approved by the joint ethics committee of the National Hospital for Neurology and Neurosurgery and the UCL Institute of Neurology and the patient gave written informed consent prior to the study onset. The patient was operated on after overnight withdrawal of levodopa medication and after dopamine agonists had been reduced and terminated during the two weeks before surgery. The DBS electrode used was model 3389 (Medtronic Neurological Division, Minneapolis, MN) with four platinum-iridium cylindrical surfaces (1.27 mm diameter and 1.5 mm length) and a centre-to-centre separation of 2 mm. Contact 0 was the lowermost and contact 3 was the uppermost. Fast acquisition T2 weighted axial and coronal stereotactic MRI scans using Leksell's Frame (Elekta, Sweden) were performed with contiguous slices of 2 mm thickness to visualize the STN and especially its medial border (Hariz et al., 2003). The anatomical target point within the centre of STN was selected in a plane at the level of the anterior border of the red nuclei on the axial image showing the largest diameter of the red nucleus (Bejjani et al., 2000). Contact 0 of the DBS electrode was targeted to this point. The double oblique trajectory to the target was planned on coronal images to avoid the ventricles. Calculations of Cartesian coordinates of the target point were performed both manually on enlarged MRI film copies and on Framelink software (Medtronic, Minneapolis, MN). No microelectrode recordings were made.

Surgery started on the right side: after infiltration of local anaesthetic, a curvilinear incision following the hair line was fashioned and a burr hole placed in line with the planned trajectory (i.e., at the level of the coronal suture, some 4 cm from the midline). A Stimloc electrode-anchoring device (Medtronic Neurological Division, Minneapolis) was applied to the skull with two mini screws and the dura and pia were coagulated and opened to allow introduction of the radio-frequency (RF) electrode. Impedance was measured to a point 5 mm above the planned target. The RF electrode was withdrawn and the DBS electrode shaft was passed down the track and advanced in 2 mm steps. Intra-operative LFP recording (Chen et al., 2006) showed a substantial rise in beta activity 3 mm above the planned target point. There was a marked introduction stun effect on rigidity and a mild effect on tremor but only a slight improvement in bradykinesia in the left hemibody. Monopolar stimulation with the electrode fixed at target produced improvement in symptoms, mostly in rigidity and tremor but also some in bradykinesia, mostly with contact zero but also in contacts 1 and 2. The electrode was fixed in position with the Stimloc device.

The procedure was repeated in the left hemisphere, beta activity rising in a more gradual fashion from 3 mm above the target level. Again the electrode was fixed at the planned target and stimulation produced a clear effect on rigidity and less on bradykinesia. Stimulation also produced a sensation of tingling in the right hand. The right electrode was tunnelled to the left incision and both electrodes were connected to an accessory kit, the connectors being tunnelled to the left temporo-parietal area and externalized to the left frontal region.

The patient received immediate post-operative stereotactic MRI using fast spin-echo T2 weighted axial and coronal MRI scans with contiguous slices of 2 mm thickness, with the Leksell frame still in situ, to confirm the location of the DBS electrodes. MRI showed good location of the electrodes in the exactly desired location. The patient was studied 3 days post-operatively, in the interval between DBS electrode implantation and subsequent connection to a subcutaneous stimulator. He was on his regular dopaminergic medication.

A healthy volunteer (29 years old, right-handed male) who consented to take part in the study performed the same task as the patient with and without percutaneous extension wires (see below) attached to his head with collodion glue.

### Experimental paradigm

The experiments with the patient and with the healthy volunteer (subsequently both called “subjects”) followed the same paradigm. The experiment was divided into blocks. During rest blocks the subjects were instructed to remain still with their eyes open for 3 min. During movement blocks the subjects performed either a “simple” movement—simultaneous button press with index, middle and ring finger or a “complex” movement sequence—index-ring-middle with either left or right hand (one kind of movement with the same hand within a block). Here, we only analyze the dominant (left) hand movements and the rest block for the patient and only the rest block for the healthy volunteer. The movements were self-initiated. The subjects were instructed to move when they felt the urge to move, but not to do it too frequently and take about 15 s between the movements without counting silently. Feedback to the subjects was presented visually, using Matlab (The MathWorks, Inc., Natick, MA) and a custom script based on the Cogent toolbox (http://www.vislab.ucl.ac.uk/cogent.php). This script monitored the movement times and displayed messages on the screen in front of the subjects when the inter-movement interval was shorter than 12 s or the “complex” sequence was incomplete. When performing correctly the subjects did not get any feedback and the screen just showed a fixation cross. In case of incorrect performance, the script waited to collect another movement so that it ran until 8 correctly performed movements were collected or at most for a total of 7 min. The subjects could usually complete a movement block in 3–4 min. Between blocks the subjects were allowed to move in the scanner and rest. A neurologist was present in the magnetically shielded room during the patient experiment to monitor the patient and performance of the task. We collected 18 blocks of data from the patient in the experiment reported here. The order of the conditions was randomized separately for blocks 1–9 and blocks 10–18; so that each half would contain one rest block and two movement blocks of each type. A single recording session lasted about 1.5–2.5 h, including preparation.

### LFP-MEG recordings

The recordings were performed with a 275 channel MEG system (CTF/VSM MedTech, Vancouver, Canada). The data were sampled at 2400 Hz and stored to disk. For subsequent off-line analysis the data was low-pass filtered at 100 Hz and down-sampled to 300 Hz. Simultaneous to the MEG signal, the LFP, electro-oculographic (EOG) and electromyographic (EMG) signals were recorded using the integrated EEG system and high-pass filtered in hardware above 1 Hz to avoid saturation of the amplifiers due to DC offsets. Four intracranial LFP channels were recorded on each side referenced to a cephalic reference (right mastoid). LFP recording was converted off-line to a bipolar montage between adjacent contacts (3 bipolar channels per side) to limit the effects of volume conduction from distant sources. EMG was recorded from right and left first dorsal interosseous (FDI) muscles with reference at the muscle tendon. One of the EMG channels on the left hand, referenced in hardware to the right mastoid, showed a clear electrocardiographic (ECG) signal and was hence also used as ECG channel.

### Data analysis

The data were analyzed using custom Matlab scripts based on SPM8 (http://www.fil.ion.ucl.ac.uk/spm/) and Fieldtrip (http://www.ru.nl/neuroimaging/fieldtrip/) toolboxes (the beamforming algorithms we used are implemented in Fieldtrip and Fieldtrip is included in the SPM8 distribution). The key analysis routines used for the present paper are freely available in the Beamforming and MEEGTools toolboxes distributed with SPM8; i.e., the SPM8 installation is sufficient to reproduce our analysis.

### MEG source analysis

Beamformer analysis was performed to (i) localize cortical sources that were coherent with STN-LFP, (ii) to compute artefact-free cortical signal using a virtual electrode approach and (iii) to localize cortical areas exhibiting movement-related power changes. The beamforming method is based on the linear projection of sensor data using a spatial filter computed from the lead field of the source of interest and either the data covariance (time domain) (Van Veen et al., 1997) or cross-spectral density matrix (frequency domain) (Gross et al., 2001).

Lead fields were computed using a single-shell head model (Nolte 2003) based on an inner skull mesh derived by inverse-normalizing a canonical mesh to the subject's individual structural image (Mattout et al., 2007). Coregistration between the MRI and MEG coordinate systems used 3 fiducial points: nasion, left and right pre-auricular points. Since the structural scan had been acquired in the clinical setting before the patient consented to take part in the study, these points were not marked with vitamin-E capsules as usually done. We, therefore, marked them by hand on head surfaces extracted from the structural image using Freesurfer (http://surfer.nmr.mgh.harvard.edu/). Subsequently, we checked these points against the original structural image and made slight correction where necessary. In order to further reduce possible coregistration error, we followed the initial rigid registration of the fiducials by an additional step, where the nasion fiducials from the two coordinate systems were brought into exact correspondence on the antero-posterior axis and the averages of the preauricular points were brought into correspondence on the superior–inferior axis. The rationale for this was that the marked nasion point was more reliable in the antero-posterior direction, as the nasion must lie on the surface of the nose, whereas the marking of pre-auricular points was more reliable in the superior–inferior direction due to the presence of clear anatomical landmarks on the ear.

Source localization was performed using the Dynamic Imaging of Coherent Sources (DICS) beamforming method (Gross et al., 2001). DICS is the optimal beamformer-based technique for localization of coherence. However, DICS filters computed for narrow frequency range are sub-optimal for extraction of virtual electrode time-series. For that we used the Linearly Constrained Minimum Variance (LCMV) beamformer (Van Veen et al., 1997). Localization of power can proceed using either DICS or LCMV. We opted for DICS since it can be combined with spectral estimation over the frequency bands of interest, using the multitaper method (Thomson 1982). This is optimal for averaging the spectral estimate over a frequency range (Mitra and Pesaran, 1999). Beamformer images were computed on a grid defined in MNI space with spacing of 5 mm and restricted to the points within the inner skull boundary. Values on the grid were then interpolated using linear interpolation to produce volumetric images with 2 mm resolution for the purposes of statistical analysis in SPM and comparison with the structural image. The resulting images were further smoothed with an 8 mm isotropic Gaussian kernel.

### Heart-beat locked artefact

For the analysis of heart-beat locked artefact, the continuous data from a rest block were high-pass filtered above 1 Hz using a 5th order zero phase Butterworth filter. QRS complexes were detected in the ECG channel using the algorithm implemented in FMRBIB plugin (Niazy et al., 2005; Christov 2004). The MEG and ECG data were then epoched from 200 ms before to 800 ms after QRS onsets.

Source time-series were extracted using Linearly Constrained Minimum Variance (LCMV) beamformer (Van Veen et al., 1997) with varying regularization (see Results). The regularization parameter was parameterized as percent of the mean of the diagonal of the channel covariance matrix. The location targeted by the beamformer was the hand area of the right primary motor cortex defined according to the literature (Mayka et al., 2006) (MNI coordinates: 37, − 18, 53). The covariance matrices for beamforming were computed from all the trials together and the source orientation was selected as the one that maximised the variance of the data explained (Sekihara et al., 2004).

### LFP-MEG coherence

For the analysis of STN-cortical coherence the continuous resting recording was divided into arbitrary epochs with a duration of 3.41 s (1024 samples). The data were high-pass filtered above 1 Hz and the line noise artefacts at 50 Hz and 100 Hz were removed using notch filters (5th order zero phase Butterworth filters). Finally, trials with artefacts in the LFP recording were rejected by thresholding the peak-to-peak LFP amplitude at 100 μV.

Sensor-level coherence was computed between one bipolar LFP channel and each MEG channel. For the present report, we chose the right STN channel showing the strongest coherence with MEG. More detailed analysis of topographical specificity of the coherence within the STN will be included in future publications. We used the multi-taper spectral estimation method (Mitra and Pesaran, 1999) in the frequency range 5 to 45 Hz with frequency resolution of 2.5 Hz.

For source coherence analysis a single DICS image was computed from all the trials of the rest block. The global maximum of this image was defined as the location of the cortical source coherent with the STN. The orientation of this source was defined as the normalized imaginary part of the cross-spectral density vector between STN-LFP and the three orientations of MEG source, located at the grid point closest to the optimal location. Typically orientation is defined by the direction of maximum power (Gross et al., 2001). We chose the imaginary part (i.e. non-zero lag) to specifically focus on physiological signals transmitted with delay between the STN and the cortex (Nolte et al., 2004) and thereby gain additional immunity from the artefact.

To further examine the activity of the cortical area coherent with the STN we performed extraction of time-series using the virtual electrode approach with LCMV beamformer (Van Veen et al., 1997) and 0.01% regularization. The covariance matrices for beamforming were computed based on the epoched data and the position and orientation of the source was as defined above.

A DICS beamformer image always has a global maximum even in cases of meaningless or erroneous localization. Therefore, we performed an additional simulation analysis to verify the internal consistency of our method. The idea was to generate a simulated coherence pattern that would be expected from the localized source and compare it with the original coherence pattern. The observed coherence pattern is determined by the location and orientation of the generating source as well as by the signal-to-noise ratios at different MEG channels or, in other words, by other cortical sources and artefacts. In our case, large metal artefacts might distort the observed coherence patterns, relative to what would be expected on the basis of source lead field alone. We, therefore, combined the simulated data with the original data to make the simulation as realistic as possible. STN-LFP data and the corresponding extracted source data were shifted by one trial with respect to the original data. The source data were then projected through the source lead field and added to the original (non-shifted data) to create simulated MEG data. We then computed the coherence between the shifted STN-LFP and the simulated MEG. Shifting eliminated the coherence between STN-LFP and the original MEG. Thus, the coherence between shifted STN-LFP and simulated MEG was solely due to the simulated component of the MEG. However, the artefacts and non-coherent brain source were the same as in the original data.

### Movement-related power dynamics

For the analysis of movement-related activations the epochs from 10 s before to 10 s after the first registered button press were used. No pre-processing was done prior to beamformer analysis. We did not distinguish between complex and simple movements for the purposes of the present analysis.

Source time-series were extracted using the LCMV beamformer (Van Veen et al., 1997) with 0.01% regularization. The source location was the hand area of the right primary motor cortex defined based on the literature (Mayka et al., 2006) (MNI coordinates: 37, − 18, 53). The covariance matrices for beamforming were computed from all the trials together and the source orientation was the one that yielded maximal variance of the data (Sekihara et al., 2004). The virtual electrode data were digitally filtered (1 Hz high pass, 48–52, 98–102 Hz notch filters, 5th order zero-phase Butterworth in all cases) and subjected to spectral analysis.

The major challenge in the analysis of patient data is that the duration of the experiment is severely limited by patient tolerance and clinical constraints; and in many cases the quality of some of the data is suboptimal. In our case, we collected 32 valid movement trials for each complexity/hand combination. This is a rather low number for event-related spectra computation. The situation would have been even worse if we had to exclude trials from analysis due to partial contamination by artefacts. We addressed this issue by combining two methods: spectral estimation using multitaper spectral analysis and use of robust averaging for computing power. Multitaper spectral analysis (Thomson, 1982) is based on pre-multiplying the data with a series of tapers optimized for producing uncorrelated estimates of the spectrum in the given frequency band. This method makes it possible to sacrifice some of the frequency resolution in a well-controlled manner to gain higher signal to noise ratio by effectively multiplying the number of trials by the number of tapers used. We estimated the spectrum in overlapping windows of 400 ms shifted by 50 ms. The frequency resolution was set to the inverse of the time window (2.5 Hz) for up to 25 Hz, then 0.1 times the frequency for 25 to 50 Hz and then to constant 5 Hz. These settings resulted in a single taper being used for 2.5–30 Hz, 2 tapers for 32.5–42.5 Hz and 3 tapers for 45 Hz and above. The resulting time–frequency images had no discontinuities in frequency thanks to the continuous frequency resolution function.

These time–frequency images were then averaged using robust averaging. This is a rather simple special case of the robust general linear model (Wager et al., 2005). The idea is that for each time–frequency pixel the distribution of values over trials is considered and the outliers are down-weighted when computing the average. This makes it possible to neutralize artefacts restricted to narrow time and frequency ranges without rejecting whole trials. Moreover, a clean average can be computed with no clean trials; given that the artefacts do not consistently overlap and only corrupt (different) parts of trials. The artefacts requiring the use of robust averaging in our case were not the metal artefacts, which were effectively suppressed by the beamformer but other artefacts such as jumps and spikes that sometimes appeared in the MEG channels. The units of average time frequency plots were converted to percentage change by normalizing to the baseline − 5 to − 3 s before the button press.

For source localization of power, DICS power images computed for specific frequency ranges and time segments around the movement (see Results for details) were subtracted and the resulting difference images were subjected to single-sample *t*-test in SPM to find the significant effects. Beamforming was applied for each block separately which resulted in 8 images per-contrast.

## Results

### Metal artefact

Prior to performing experiments with patients we assessed the artefact induced in the MEG signal due to the presence of the DBS electrode by holding the electrode inside the MEG helmet and by recording auditory evoked fields in a healthy subject with DBS electrodes attached to the subject's head (data not shown). This did not reveal any effect of the DBS electrode on the MEG signals. However, in experiments with patients we observed high-amplitude deflections completely obscuring the physiological signal for some of the channels, particularly those close to the burr holes. Closer examination revealed that these artefacts originated from the percutaneous extension wire made of stainless steel (Lynn Otten, Medtronic, personal communication). The actual DBS electrode is made of titanium and is therefore not ferromagnetic and does not interfere with the MEG recording, as we initially observed. The stimulating end of the electrode is embedded in the STN during the first surgery. The other end (sticking out of the brain) is subsequently guided under the skin to the chest and connected to a subcutaneous stimulator during a second surgery. Between the two surgeries this part of the electrode should remain sterile and it is therefore wound up and placed under the scalp. In order to enable stimulation and recording during this period an additional wire is connected to the sterile electrode under the skin and its other end is externalized. This stainless steel percutaneous extension wire is later discarded.

**Fig. 1:**
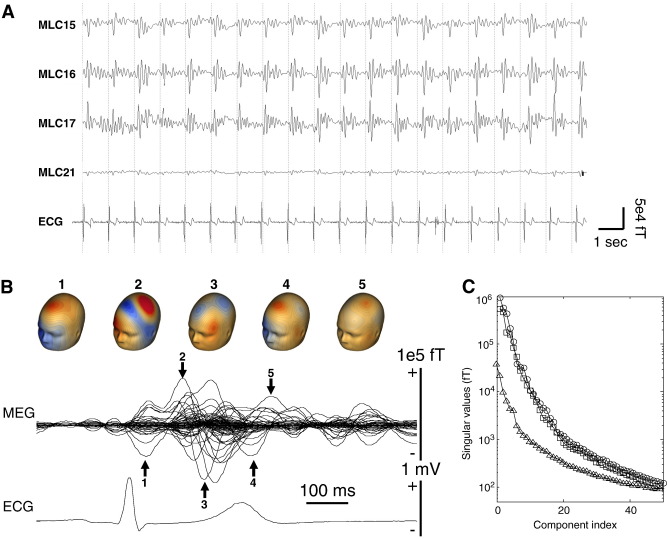
Properties of the metal artefact seen in the MEG of DBS patients. (A) Raw data (high-pass filtered above 5 Hz) from several MEG channels close to the burr hole showing artefact deflections following the peaks of the ECG. The artefact exceeds the typical physiological signal amplitude by several orders of magnitude. (B) QRS-locked average of the MEG (all channels shown together) and the ECG. Numbered arrows point at some of the prominent artefact peaks and the corresponding MEG scalp maps are shown above the trace. The topographies vary greatly between peaks and could not be explained by a single source. (C) Plot of the singular values for decomposition of the QRS-locked average shown in B (circles) shown with the singular values from the same analysis performed on healthy volunteer data with (squares) and without (triangles) percutaneous extension wires glued to the subject's head. The artefact in the healthy subject was similar in magnitude to that of the patient (circles). For both the patient and the healthy subject there were about 6 singular values exceeding the maximal value observed without wires. This reflects the complex spatial topography of the artefact also seen from the scalp maps in B.

We tested the percutaneous extension wire by moving it in the MEG helmet and recorded from a healthy volunteer with two extension wires attached to his scalp in positions typically seen in patients, using collodion glue. In both cases we observed severe artefacts in the MEG signals. Fig. 1 illustrates the artefact properties. The most affected channels were at the front close to the burr holes. In this particular patient both the left and the right wire were externalized on the left side, which decreased the artefact on the right but increased it on the left, relative to the more common situation when each wire is externalized on its respective side. In the left channels, the artefact amplitude reached 100 pT—orders of magnitude higher than typical physiological signal amplitude. The artefact deflections repeated with a periodicity of about 1 s in both the DBS patient and the healthy volunteer with wires. By comparing the timing of the deflections with ECG peaks, we concluded that the artefact deflections were highly correlated (Fig. 1A). This was confirmed by averaging MEG around the times of ECG peaks. In the ensuing ECG-triggered average of the MEG signal (Fig. 1B) the strongest artefact deflections appear between 200 and 700 ms after the QRS pulse in the ECG, the time corresponding to the ejection phase of the cardiac cycle. The changes in artefact topography during this period are characteristic of a rotating magnetic source rather than a change of amplitude of a single topography. This explains why singular value decomposition (SVD) of the cardiac event-related field (ERF, Fig. 1C) results in about 6 singular values above the highest value in SVD of a metal artefact free signal recorded from a healthy subject. In the case of the healthy subject with percutaneous extension wires attached to the scalp the artefact amplitude was similar to that of the patient and with a similarly complex topography, as seen from the SVD.

### Suppression of the artefact using beamformer

**Fig. 2:**
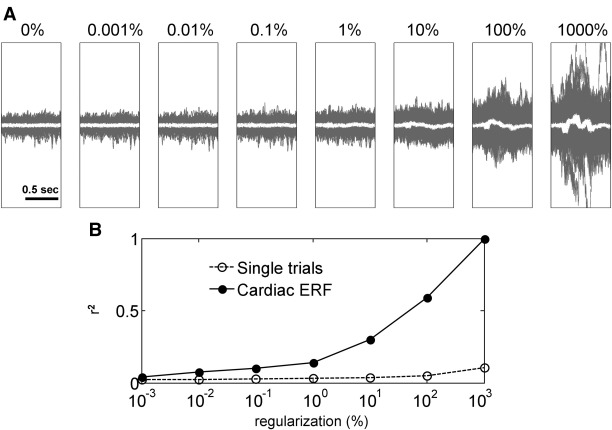
Beamforming suppresses the metal artefact. Virtual electrode of LCMV beamformer was placed at the right M1 (MNI coordinates: 37, − 18, 53) and source activity was computed for single trials defined around heart beats. Regularization parameter of the beamformer (defined as percentage of the mean of the diagonal of the channel covariance matrix) was varied systematically over the levels depicted. (A) Single trial source waveforms (grey) and the average across trials (white) for different levels of regularization. For very high regularization (when the beamformer has low spatial selectivity) the source data are heavily contaminated by the heartbeat-locked artefact. With decreasing the regularization the cardiac-locked activity is substantially diminished. (B) To quantify the artefact contamination we computed the *r*^2^ value for correlation between the average cardiac-locked source waveform for regularization of 1000% (white trace in the rightmost plot of A) and single trials and averages of the other plots. In the case of single trials the trial-specific *r*^2^ values were then averaged. This analysis also shows that decrease in artefact contamination is most dramatic between 1000% and 1% regularization and continues with further decreases in regularization.

In order to demonstrate that the artefact can be effectively suppressed using beamforming we used the virtual electrode approach to extract source time-series from the right M1 in the presence of artefact. We considered a situation more extreme than our typical analysis by epoching the data around the onsets of QRS complexes in the ECG. We then varied the regularization parameter of the LCMV beamformer (parameterized as percentage of the mean of the diagonal of the channel covariance matrix). By varying the regularization parameter it is possible to make the beamformer filter more spatially selective for lower regularization values or less spatially selective (and close to pseudo-inverse of the source lead field; i.e. ordinary dipole waveform estimation) for high regularization values (Brookes et al., 2008b). From the rest block of the patient recording 188 trials were extracted. The average source waveform for the highest regularization value tested (1000%) was dominated by artefact and taken as the artefact template. Correlation with this template was then tested for single trials and averages for 8 regularization levels: 0%, 0.001%, 0.01%, 0.1%, 1%, 10%, 100% and 1000%. The results are presented in Fig. 2. Decreasing regularization resulted in substantial suppression of the artefact (after regularization of 1%) as evidenced by low *r*^2^ values for the correlation with artefact template (Fig. 2B) and absence of strong QRS locked deflections in single trials and in the average (Fig. 2A). Further decrease of regularization yielded further suppression of the artefact, but the improvement was relatively minor. Therefore, we concluded that regularization can be set in the range from 0% to about 1%, without compromising artefact suppression.

### Localization of LFP-MEG coherence

**Fig. 3:**
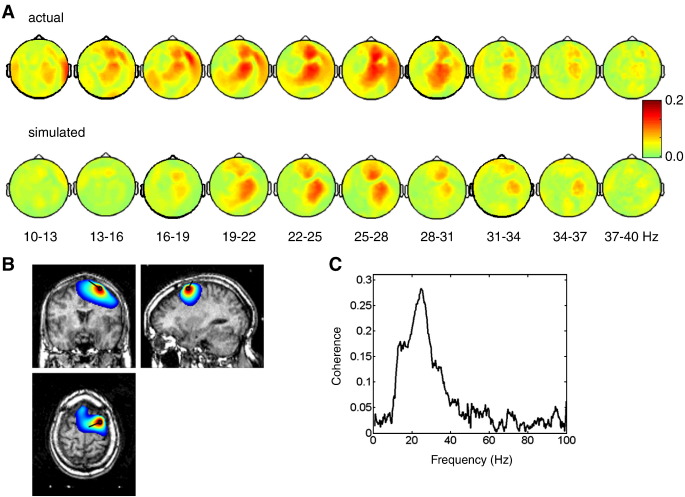
Analysis of the coherence between cortex and STN using beamforming. (A) Scalp topography of the coherence between the right STN bipolar channel showing the strongest coherence with the MEG and the MEG sensors for different frequency ranges. The top row shows the original coherence and the bottom row shows coherence patterns simulated assuming that the cortical source coherent with the STN was the single dipole shown in B. (B) DICS beamformer source reconstruction for the coherence pattern shown in the top row of A. Channel cross frequency matrix was computed for 15 to 35 Hz range. The resulting beamformer image was thresholded at mean + 4 times the standard deviation across voxels and overlaid on the subject's structural image. An equivalent current dipole was placed at the global maximum of the beamformer image (MNI coordinates: 28, 4, 66) and its orientation was defined as normalized imaginary part of the cross-spectral density between the STN signal and the 3 spatial orientations of the MEG. (C) Virtual electrode time-series were computed using LCMV beamformer for the dipole source shown in B. The coherence spectrum between these time-series and the STN signal is shown. The peak coherence was 0.28 at 25 Hz, which is quite high for neural signals. The maximum and the width of the coherence peak are consistent with the scalp coherence pattern in A.

Topographical mapping of LFP-MEG coherence revealed frequency-specific patterns consistent with physiological cortical generators. Despite heavy contamination of the MEG signal with metal artefacts as described above, these patterns were obtained with very little pre-processing of the data. Fig. 3A (top row) shows the coherence patterns for the right STN channel in the patient presented here. The highest coherence was between 15 and 35 Hz. The coherence was clearly lateralized to the right. We applied DICS coherence beamformer for localization of the cortical area coherent with the STN. Fig. 3B shows the beamformer image thresholded for visualization purposes at mean plus 4 times the standard deviation (computed across voxels) and superimposed on the patient's structural image. The global maximum of the image was at MNI coordinates: 28, 4, 66. Virtual electrode time-series extracted from this source were coherent with the STN signal in the frequency range consistent with sensor-level coherence (Fig. 3C). To demonstrate that this source is a plausible candidate generator for the coherence pattern observed at the sensor level, we compared the observed coherence pattern with a simulated coherence pattern generated by projecting extracted source time-series via the lead-field and adding it to the original MEG data to have the same metal artefact and other confounds (see Methods and Fig. 3A, bottom row). The simulated coherence pattern was consistent with part of the original pattern. There were also features in the original pattern not explained by this dipole, indicating the existence of additional coherent sources.

### Analysis of movement-related power

**Fig. 4:**
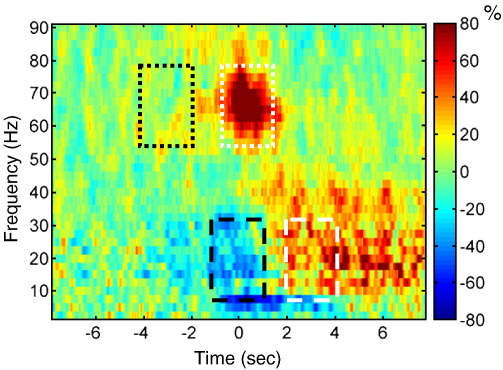
Power changes around self-initiated left finger movements. Virtual electrode was placed at the right M1 (MNI coordinates: 37, − 18, 53) and time-series were extracted around the times when the subjects performed button presses with the fingers of his left hand. Time–frequency decomposition of the time-series shows ERD/ERS sequence in the alpha and beta bands and ERS in the high gamma band, which are well known features of movement-related cortical power dynamics. The rectangles show the time and frequency ranges that were used for volumetric beamformer analysis shown in Fig. 5. The dotted line corresponds to the high gamma range (55–80 Hz) and the dashed line to alpha and beta range (5 to 30 Hz). In the subtraction between two time windows the images corresponding to white rectangles entered with the positive sign and the images corresponding to black rectangles with the negative sign.

**Fig. 5:**
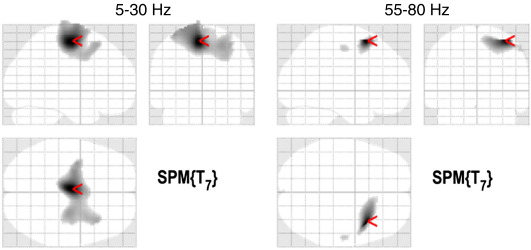
Volumetric DICS beamformer source reconstruction of cortical areas undergoing movement-related power changes. The time and frequency ranges were as shown in Fig. 4. Single-sample *t*-test was performed on 8 difference images computed for each movement block. The *t*-statistic was thresholded at the level corresponding to *p* < 0.05 (family-wise error corrected). Mesial and contralateral motor and pre-motor areas were revealed. The peak power difference was at − 4, − 10, 62 mm for the low frequency range and at 36, 16, 62 mm for the high frequency range.

We used the virtual electrode approach to extract time-series from the hand area of the right M1. Sixty four self-initiated button presses were analyzed (both "complex" and "simple"). Fig. 4 shows the average time–frequency around a button press with the left hand. Event related desynchronisation (ERD) can be observed in the alpha and beta bands starting around 1 s before the movement, followed by event related synchronization (ERS) from about 2 s after the movement. At the time of the movement, high-gamma ERS can be observed from about − 0.5 to 1.5 s relative to the button press. We used the DICS beamformer to localize areas exhibiting similar power dynamics. For the lower frequency range we subtracted the images of power in 5–30 Hz for − 1 to 1 s from the images for the same frequency range for 2 to 4 s relative to the button press. For the higher frequency range we subtracted images for 55 to 80 Hz and − 4 to − 2 s from images for the same frequency range and − 0.5 to 1.5 s relative to the button press. Beamformer filters were computed separately for each block and the power differences were averaged across trials in the block. Eight images computed in this way were subjected to single-sample *t*-test with *p* < 0.05 family-wise error correction. The results are shown in Fig. 5A (lower frequency range) and 5B (higher frequency range). In both cases, the significant power changes localized to the hemisphere contralateral to the movement with peaks in the sensorimotor area.

## Discussion

Our results confirm the hypothesis that beamforming is particularly suitable for suppressing artefacts that confound MEG recordings of DBS patients. We were able to optimize the degree of regularization and identify a sufficient range of regularization for artefact suppression. We then went on to establish the face-validity of beamforming in this context by showing that source activity reconstructed using data confounded by artefacts showed canonical time–frequency correlates of movement-related activity. Furthermore, we were able to show the predictive validity of these estimates, in terms of their reproducibility over independent realizations of time–frequency responses (different recording blocks).

The utility of beamforming rests on the fact that the artefacts cannot be explained by localized cortical sources. We have shown that the artefact in MEG recordings of DBS patients is generated by the percutaneous extension wires, which leads to a high-dimensional and complicated topographic expression. Beamforming is particularly robust to this sort of artefact (Brookes et al., 2008a).

The actual DBS electrode is not ferromagnetic and is, therefore, MEG-compatible. The use of percutaneous extension wires distinguishes DBS patients and epilepsy patients with intracranial electrodes. In the case of epilepsy, an intracranial electrode is implanted for a short period and its external end does not have to be kept sterile, since it will be removed eventually. It is, therefore, externalized directly without an extension wire. Indeed, several research groups have reported simultaneous intracranial LFP and MEG recordings in epilepsy patients and these reports do not mention severe artefacts in the MEG, such as we observed (Dalal et al., 2009; Santiuste et al., 2008). This means that the major technical problem presently hampering simultaneous LFP-MEG recordings in DBS patients could possibly be resolved by using percutaneous extension wires made of non-ferromagnetic material such as titanium. The manufacturers may be motivated to develop such wires in the future, if simultaneous LFP-MEG recordings are found useful for clinical purposes. Presently, however, it is not possible to replace the ferromagnetic wire with a different wire since the manufacturers would be unlikely to accept responsibility for the functioning of the system in a clinical setting.

Another possible source of artefact might be small metal particles originating from the screws that are left in the bone after drilling the burr hole. We have not found evidence that this is a major problem. One of the patients in our series suffered an absence attack following implantation of a DBS electrode on one side and, as a result, she was implanted unilaterally on the right although burr holes were drilled on both sides. In this patient there was little artefact on the left, although it is hard to assess how much of it would still be present with no wires at all. Evidence from epilepsy patients suggests that in any case such an artefact could at worst make the recordings slightly noisier but would not require special analysis techniques of the sort proposed here.

We also observed that the artefact is to a large extent time-locked to the heart beat. The spatial topography of the artefact is quite complex and has a 'rotating' pattern within the heartbeat cycle, quite similar to the ballistocardiogram artefact observed in simultaneous EEG-fMRI recordings. Therefore, the properties of the two kinds of artefacts might have similar underlying mechanisms. Debener et al. (2008) proposed that the ballistocardiogram artefact is generated by both pulsation of the blood vessels and minor head movements that occur during the cardiac cycle. Both mechanisms might apply to our MEG artefact. One of the consequences of the rotating topography of the artefact is that it cannot be adequately represented by a small number of topographical components and is, therefore, difficult to remove using topography-based artefact correction methods such as principal component analysis (PCA) or independent component analysis (ICA). In the case of the ballistocardiogram, more sophisticated versions of these methods have been shown to be quite effective, but for MEG there is a further complication: the sensor positions in relation to the head are not fixed, particularly in patients who find it more difficult to keep still than healthy subjects. We have not attempted to use ICA on our data, but we have tried the topography-based method proposed by Berg and Scherg (Berg and Scherg, 1994). The artefact could be corrected with a large number of PCA components (more than 10). However, the use of this method has substantial effects on the coherence topographies, probably due to linear mixing of coherent and non-coherent channels. Nevertheless, PCA-based or other de-noising might be useful as part of the analysis stream.

The Signal Space Separation (SSS) approach has been proposed by the developers of the Neuromag MEG system for artefact removal (Taulu and Simola, 2006). This approach is based on separating sources within the brain from sources outside the brain based on their expected effect on the MEG sensors. A further extension of this approach, temporal SSS (tSSS), also includes an additional step of regressing out, in the time domain, the signals that can be explained by the artefact sources from the brain activity (Medvedovsky et al., 2007). This approach has been reported as effective for de-noising evoked fields recorded from patients with chronically implanted DBS stimulator, even when stimulation was on (Makela et al., 2007). We could not test this method as it is not implemented for the CTF system. The chronically implanted patients differ from our patients since they have a stimulator in their chest and do not have percutaneous extension wires. It is, therefore, difficult to say at this stage how effective SSS would be for simultaneous LFP-MEG recordings. The virtual electrode signals obtained with beamforming alone appear to be sufficiently artefact-free. However, the spatial resolution of source localization could possibly be improved by combination with SSS (Vrba et al., 2009). Sensor-level de-noising could also make other kinds of analysis possible such as analysis of evoked fields.

Another possible approach for artefact removal is modelling the artefact with an explicit generative model and including this model in the analysis, such as in electromagnetic source reconstruction or dynamic causal modelling (Kiebel et al., 2009). This approach is theoretically appealing, but would probably be difficult to implement due to the complex shape of the wires and multiple factors influencing their movement. The great advantage of the beamformer is its ability to suppress artefacts in an adaptive way without an explicit generative model.

The application of beamforming for metal artefact suppression in a clinical setting was first demonstrated by Cheyne et al. (2007). These authors used LCMV beamformer for localization of event-related fields in patients with dental implants for pre-operative functional mapping. They reported source localization accuracy comparable to what could be achieved in patients without metal implants. Our work affirms the results of Cheyne et al. and extends them in several ways. First, the metal artefact in DBS patients is even more severe than in subjects with dental implants; due to the wires being partially subcutaneous, much closer to the MEG sensors and affected by the heartbeat. The artefacts generated by dental implants have been attributed by Cheyne et al. to small vibrations and undetected motions of the jaw. In contrast, the artefacts locked to heartbeat in DBS patients affect the recording continuously to the extent that it needs to be demonstrated that brain signals can still be extracted from the data. Establishing this was one of the main objectives of this work. Secondly, Cheyne et al. performed source localization but have not shown that artefact-free virtual electrode signal can be extracted from the sources. This is very important for our work as (at least in the case of movement-related activity) the generators are quite well known and it is more important to look at the interplay between the cortex and STN during movement preparation and execution. This will be the focus of future publications. Thirdly, we extended the results of Cheyne et al. to beamforming in frequency domain, critically, to localizing sources coherent with the STN. This is a crucial aspect of simultaneous MEG-LFP recordings: the ability to localize coherent sources is one of the main advantages of using MEG rather than EEG and simultaneous rather than separate recordings.

Coherence between STN-LFP recorded as potential difference between adjacent STN contacts and MEG signals is a quite robust phenomenon and can be seen with practically no pre-processing of the data. The reason for this is the existence of consistent phase relations between the artefact-free STN-LFP signal and some physiological cortical signals and the absence of such consistent phase relations between the STN-LFP signal and the artefact. There are several reasons to believe that the coherence patterns we observed are not artefacts. The frequency range in which coherence was observed is consistent with that of previously reported LFP-EEG coherence (Lalo et al., 2008; Fogelson et al., 2006; Cassidy et al., 2002; Williams et al., 2002). In the subject presented here, the coherence lateralized to the side of the corresponding STN. Furthermore the MEG channels most affected by the artefact are the frontal channels close to the wires. One would expect that presence of high amplitude noise not coherent with the STN would reduce LFP-MEG coherence and this indeed seems to be the case for these channels. However, lateralized patterns were observed at central MEG channels, which are less affected by the artefact.

Consistent phase relations between LFP and MEG signals revealed by coherence can be exploited by a number of analysis methods to localize the cortical sources coherent with the STN and to study their properties. One of these methods is the DICS beamformer that we use in the present paper (Schnitzler and Gross, 2005). One can also use methods based on phase-alignment (Guderian and Düzel, 2005; Lehmann and Michel, 1990; Lütkenhöner 1992) to selectively average the signals phase-locked to the STN-LFP and average out the artefact. Also methods based on linear prediction such as vector auto regressive (VAR) modelling effectively exploit this kind of consistent phase relation and can be applied to MEG-LFP data in the presence of artefacts. However, an important point to remember when applying these methods is that most of them are based on some kind of averaging. When the artefact exceeds the physiological signal of interest by several orders of magnitude, it is not always reduced sufficiently by averaging. Thus, in practice, methods based on phase consistency should be combined with at least coarse correction of the artefact based on its spatial topography; for instance, using SVD. DICS beamforming is appealing in this sense because it implicitly exploits both spatial topographies of different sources and the phase relations between them. To our knowledge, this is the first time the orientation of the DICS beamformer has been estimated using the imaginary coherence components.

We have demonstrated that LCMV and DICS beamformers can be used to effectively suppress the artefacts in combined LFP-MEG recordings, to localize cortical sources based on their coherence with the STN or on specific power dynamics in a task and to extract artefact-free source signals that can be further analyzed with any standard multivariate time-series analysis methods. For practical applications it is important to optimize the value of the regularization parameter. Excessive regularization can result in insufficient suppression of the artefact, whereas some regularization is necessary especially when looking at single trials or combining trials with slightly different head positions (Brookes et al., 2008b). Also, when using source locations based on the literature, the actual sources of interest can be missed with no regularization due to inter-subject differences (Barnes et al., 2004). Based on our results, there appears to be quite a wide range of regularization parameter values for which the artefact is effectively suppressed, making it possible to adapt the regularization based on the amount and quality of the data.

The power dynamics around movement found in the patient in the presence of artefact (Fig. 4) were consistent with the ERD/ERS pattern previously described in the literature based on EEG and MEG recordings in healthy subjects, electrocorticography (ECoG) recordings in epilepsy patients and STN-LFP recordings in patients with PD (Pfurtscheller and Lopes Da Silva, 1999; Kempf et al., 2007; Androulidakis et al., 2007; Miller et al., 2007; Crone et al., 1998a;b; Alegre et al., 2005; Jurkiewicz et al., 2006, Cheyne et al., 2008). In terms of the spatial locations of the contrast peaks (Fig. 5) the beta ERD/ERS is bilateral as in the MEG study of Jurkiewicz et al. (2006), but not limited to the two symmetric hand areas; and the peak is actually in the mesial motor area. The high gamma peak is anterior to the location reported by Cheyne et al. (2008). This can be attributed partly to the conservativeness of family-wise correction. With lower statistical threshold beta ERD/ERS can be seen in the ipsilateral hand area as well. In addition, there were differences in details of the task. The subjects in Jurkiewicz et al. (2006) and Cheyne et al. (2008) study performed brisk and frequent simple index finger movements, whereas our subjects performed infrequent movements where they completely controlled the timing. Half of the movement were complex sequences. Under these circumstances one might expect greater involvement of the Supplementary Motor Area (SMA), which would be consistent with the mesial activations we observed. Finally, an obvious explanation is the difference between healthy subjects studied previously and a patient with advanced PD, whose motor representations might have undergone extensive plastic changes as a direct result of the disease and following compensatory motor learning.

The beamformer method is flexible enough to effectively solve the artefact problem. It makes it possible to fully characterize task-related power and coherence dynamics in the presence of artefacts given some starting point. For instance, knowing at least one cortical area that is involved in a task, one can extract the source activity from this area, characterize the power dynamics and then scan the whole brain to find areas displaying similar dynamics and proceed iteratively. Similarly, one can start with some prior knowledge about the power dynamics of interest, find the brain areas displaying these dynamics, extract source activity from these areas and characterize the power dynamics in greater detail. Although we focused on PD patients with electrodes in the STN, our methods are applicable to any group of patients with intracranial electrodes and indeed to other group of subjects where artefacts are a problem (as an extension of approach of Cheyne et al. (2007)). The ability to simultaneously record intracranial local field potentials and MEG should provide significant insight into cortico-cortical and subcortico-cortical interactions in neurological disease in the future.
